# Towards plug-and-play integration of archetypes into legacy electronic health record systems: the ArchiMed experience

**DOI:** 10.1186/1472-6947-13-11

**Published:** 2013-01-22

**Authors:** Georg Duftschmid, Judith Chaloupka, Christoph Rinner

**Affiliations:** 1 Section for Medical Information Management and Imaging, Centre for Medical Statistics, Informatics and Intelligent Systems, Medical University of Vienna, Vienna, Austria

**Keywords:** Medical records, Medical records systems, Computerized, Reference standards, Models, Theoretical

## Abstract

**Background:**

The dual model approach represents a promising solution for achieving semantically interoperable standardized electronic health record (EHR) exchange. Its acceptance, however, will depend on the effort required for integrating archetypes into legacy EHR systems.

**Methods:**

We propose a corresponding approach that: (a) automatically generates entry forms in legacy EHR systems from archetypes; and (b) allows the immediate export of EHR documents that are recorded via the generated forms and stored in the EHR systems’ internal format as standardized and archetype-compliant EHR extracts. As a prerequisite for applying our approach, we define a set of basic requirements for the EHR systems.

**Results:**

We tested our approach with an EHR system called ArchiMed and were able to successfully integrate 15 archetypes from a test set of 27. For 12 archetypes, the form generation failed owing to a particular type of complex structure (multiple repeating subnodes), which was prescribed by the archetypes but not supported by ArchiMed’s data model.

**Conclusions:**

Our experiences show that archetypes should be customized based on the planned application scenario before their integration. This would allow problematic structures to be dissolved and irrelevant optional archetype nodes to be removed. For customization of archetypes, openEHR templates or specialized archetypes may be employed. Gaps in the data types or terminological features supported by an EHR system will often not preclude integration of the relevant archetypes. More work needs to be done on the usability of the generated forms.

## Background

According to the EHR IMPACT study, interoperability is a key factor for the success of electronic health record (EHR) systems [1]. In today’s heterogeneous world of health information technology with many different EHR systems on the market, the employment of EHR standards is widely seen as a prerequisite for interoperability [2-4]. In this scenario, EHR systems transform the data to be exchanged from their internal format to a common standard format called *EHR extract*[5] and vice versa. To obtain optimum information management for integrated care, semantic interoperability should be strived for [5].

The dual model approach represents a promising method for achieving semantic interoperability [6]. It combines two kinds of models, the *Reference Model* (RM) and *Archetype Model* (AM), to represent EHR content [7]. By specifying the structure of an individual EHR content and providing an interface to medical terminology, archetypes are an important means of achieving semantic interoperability. Currently, ISO/EN 13606, HL7 Clinical Document Architecture, and openEHR represent the most important dual model based EHR standards [8-10]. HL7 is currently working on the so-called *templates* concept [11], which is conceptually similar to archetypes. In the following, we refer only to archetypes.

A frequently stated benefit of the dual model approach is that, unlike the single model approach, EHR systems do not have to be programmatically updated each time new types of EHR content have to be introduced or existing ones need to be modified [4,7,12]. In the dual model approach only the stable RM is “hardcoded” in the EHR system. Modifications of existing and additions of new archetypes can be handled without having to reprogram the EHR system, as shown in several pilot implementations of the dual model approach [13-15].

Existing implementations typically require some sort of manual system parameterization when integrating an archetype, such as a manual mapping between the archetype and the internal data model of the EHR system. If the effort involved in this system parameterization exceeds a certain limit, the dual model approach will still not be practicable. The ideal solution would be automatic integration of archetypes into an EHR system without any manual effort. This corresponds to the so-called “plug-and-play” integration of archetypes in [12].

Previous work on plug-and-play integration of archetypes focused primarily on the automatic generation of forms from archetypes within EHR systems where the latter are already internally based on a dual data model [16-19]. In contrast to these, our present work concentrates on the integration of archetypes into legacy EHR systems with proprietary internal data models. In accordance with [9], we assume that the dual model approach is used only to standardize the communication layer. This complicates the task insofar as the limitations of the legacy EHR system data models have to be considered.

In [20] Chen et al. present an approach for an automatic bi-directional conversion between openEHR archetypes and the internal data model of an EHR system called COSMIC. They describe how the AM and RM can be semantically mapped to so-called COSMIC templates, which can be directly used to record data within the COSMIC system.

Our goal is to extend the work of Chen et al. with respect to the following:

Based on their semantic mapping, we develop a more generalized approach for automatically generating entry forms in legacy EHR systems from archetypes. As a prerequisite for applying our approach, we define a set of basic requirements for the EHR systems, which are in accordance with ISO/TS 18308 “Requirements for an Electronic Health Record Architecture” [21].

Additionally, we introduce a method for the immediate export of EHR documents that are recorded via the generated forms and stored in the EHR systems’ internal proprietary format as standardized and archetype-compliant EHR extracts.

To test our approach, we implemented a corresponding prototype within the EHR system ArchiMed [22].

We chose the openEHR architecture for our study, as it currently provides the most mature public library of archetypes [23].

In this study, our focus is on the integration of archetypes into legacy EHR systems, where the archetypes have been published by an organisation that adheres to the principles of domain knowledge governance [24]. Therefore, we do not address the transformation of EHR system forms into archetypes.

## Methods

**Figure 1:**
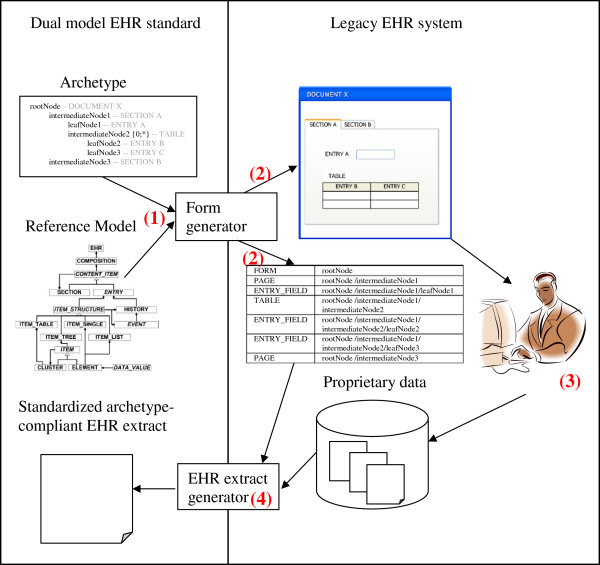
**Plug-and-play integration of archetypes into legacy EHR systems.** In step (1), the form generator, using existing tools [25], parses the archetype formatted in the Archetype Description Language, which is shown here in a pseudo notation for reasons of space and simplicity. It then augments the archetype with the implicit constraints from the RM to create a comprehensive archetype. This allows attributes of the RM, which are not constrained in the archetype, to be included in the generated form. (2) From the comprehensive archetype, the EHR system form and a mapping table are generated. For each form component, the latter stores the path of its source archetype node. (3) The generated form is used to record documents and store them in the internal format of the EHR system. (4) The EHR extract generator uses the generated mapping table to transform documents from their internal format to an EHR extract that is compliant with the archetype and RM.

In the following, we first address the requirements that must be satisfied by an EHR system’s data model as a prerequisite for applying our approach for plug-and-play integration of archetypes. We then describe the first part of our approach, i.e., the automatic generation of entry forms within legacy EHR systems from archetypes (cf. Figure 1). Finally, we explain the second part of our approach, that is, how EHR documents that are recorded via the generated forms and stored in the EHR system’s internal format may immediately be exported as standardized and archetype-compliant EHR extracts.

### Prerequisites for applying our approach

We restrict our prerequisites to a small number of basic requirements to enhance the general applicability of our approach. The following requirements for an EHR system’s data model that are needed in order to apply our approach are supported by corresponding statements in the ISO/TS 18308 “Requirements for an Electronic Health Record Architecture” [21]:

● It must contain a component that represents entry forms. This is supported by ISO/TS 18308 requirement PRO1.1: “The EHR architecture shall support the recording of any type of clinical event […] relevant to the care of a patient”, insofar as clinical events are typically recorded via forms in an EHR system.

● It must contain a component that represents labelled entry fields. This demand is supported by ISO/TS 18308 requirement STR2.4: “The EHR architecture shall enable storage of data such that simple name/value pairing is preserved”.

● It must support a dynamic duplication of entry fields during documentation (e.g., via tables with extendable rows). This is essential for the representation of repeating archetype nodes, i.e., nodes with an upper *occurrence* limit greater than 1. This demand is supported by ISO/TS 18308 requirement STR2.2: “The EHR architecture shall enable storage of data in tables such that the relationships of data with the row and column headings are preserved”.

● It should support at least textual, numeric, date, and time data types. This demand is supported by ISO/TS 18308 requirements STR2.6: “The EHR architecture shall support the inclusion of narrative free text”, STR3.1: “The EHR architecture shall support the definition of the logical structure of numeric and quantifiable data […]”, and STR3.6: “The EHR architecture shall support the definition of the logical structure of dates and times”. The closer an EHR system’s set of supported data types matches the set of data types used in archetypes, the smaller is the loss of data quality when transforming an archetype to an EHR system form.

The EHR system must further allow individual access of all form components and all data recorded via forms. Depending on the underlying database, SQL queries, XQueries, or similar technologies may be applied for this purpose.

### Automatic generation of EHR system forms from archetypes

**Figure 2:**
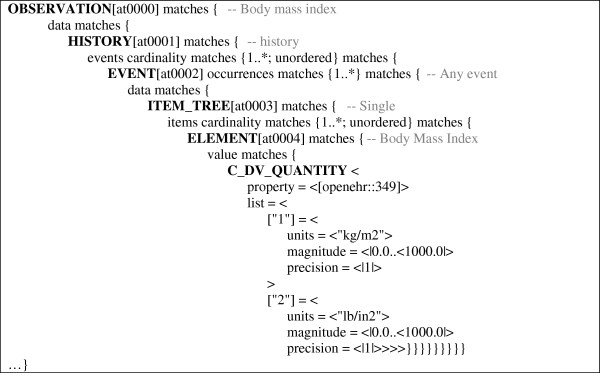
**Excerpt of the definition section of archetype “openEHR-EHR-OBSERVATION.body_mass_index.v1”.** Local terms of archetype nodes are depicted as greyed comments. The definition section describes the tree-like structure of archetype nodes from which the EHR system form is derived.

An openEHR archetype (see Figure 2) consists of a tree-like hierarchical structure of nodes, which define valid instantiations of the openEHR RM. Each node constrains a class of the RM or a data type. Archetype leaf nodes constrain a primitive data type. The data that are to be collected in the generated EHR system form are exclusively described by the leaf nodes. All other nodes serve to describe the structural and semantic context of the data to be collected.

For the generation of EHR system forms from archetypes, the latter have to be augmented to *comprehensive archetypes*[15] in the first step. This is essential as archetypes only include those constraints, which they tighten with respect to the RM. Mandatory attributes of the RM that are not further constrained by the archetype can be seen as “implicit” constraints, which also have to be considered and must be “looked up” in the RM. As an example, the openEHR RM prescribes a mandatory attribute *origin* for class HISTORY, which is not addressed in node *at0001* of Figure 2, but still has to appear in the generated form. When creating the comprehensive archetypes, the archetypes are augmented with the implicit constraints. In the following steps, comprehensive archetypes are used exclusively.

EHR system forms may be derived from archetypes based on a three-layered semantic mapping that addresses structural constraints, data value constraints, and terminology related constraints [20].

### Structural constraints mapping

The goal of this step is to map the hierarchical structures of archetype nodes to semantically comparable structures within the EHR system data model.

The entry points in the two models, which are mapped to each other, are the archetype root node and the EHR system form. Semantically, an archetype node of class COMPOSITION represents an obvious counterpart of the EHR system form, as both describe the structure for a class of documents. However, archetypes frequently start with a root node that resides below the COMPOSITION class in the RM hierarchy. In this case, the form that is derived from the root node may be seen as an artificial container that is required in the EHR system to document the data described by the archetype.

**Figure 3:**
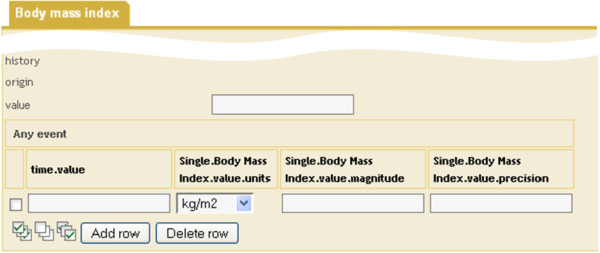
**Excerpt of ArchiMed form derived from archetype “openEHR-EHR-OBSERVATION.body_mass_index.v1” shown in Figure**2**.**

Leaf nodes may be mapped to entry fields. Intermediate nodes describing the context of “their” leaf node may be mapped to textual labels, which precede the label of the entry field (e.g., compare column “Single. BodyMassIndex.value.units” in Figure 3). Naturally, the nodes’ local terms as defined in the archetype ontology section can be the source of the labels. In Figure 2 the local terms are shown as comments of the corresponding nodes.

If the EHR system supports additional “organisational” form components (e.g., pages, sections, or groups) corresponding to the semantics of RM classes, the context may alternatively be expressed by mapping the intermediate nodes to these form components.

Repeating archetype nodes, i.e., nodes with an upper *occurrence* limit greater than 1 (such as node *at0002* in Figure 2), must be mapped to a form component, which allows entry fields to be dynamically duplicated during documentation (e.g., a table). The leaf nodes of the different branches “below” the repeating node represent the entry fields, which may be dynamically duplicated.

**Figure 4:**
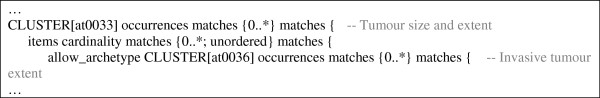
Excerpt of the definition section of archetype “openEHR-EHR-CLUSTER.microscopy_breast_carcinoma.v1.adl”, which specifies two levels of repeating nodes.

EHR system data models do not usually support a *recursive* duplication of entry fields during documentation. Thus, the mapping will fail if an archetype includes multiple levels of repeating nodes, i.e., a repeating node holding a repeating subnode (cf. Figure 4).

**Figure 5:**
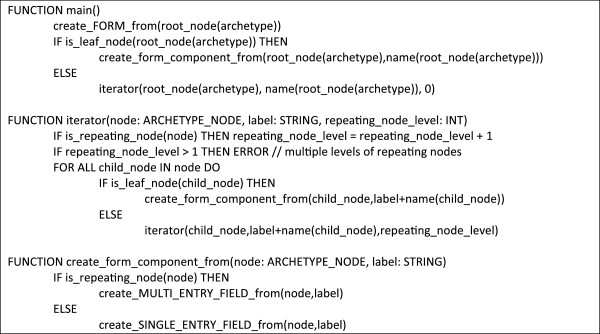
**Algorithm in pseudo-code for mapping the structural constraints of archetypes to semantically comparable structures within the EHR system data model.** The algorithm starts with function *main()*. It has access to the variable *archetype*, which holds the comprehensive archetype. Function *create_FORM_from()* creates a new form within the EHR system. Function is_leaf_node() tests whether a node is a leaf node of the archetype hierarchy, i.e., does not hold subnodes. Function *is_repeating_node()* tests whether a node has a greater maximum occurrence than 1. Function *create_MULTI_ENTRY_FIELD_from()* creates an entry field for a leaf node that may be dynamically duplicated during documentation and associates it with a label that integrates the names of all parent nodes to make the entry field’s context obvious. It further stores the complete path of the archetype leaf node, which is later used for the creation of EHR extracts from the collected data. Function *create_SINGLE_ENTRY_FIELD_from()* does the same for a single entry field.

Figure 5 summarizes the structural constraints mapping using pseudo-code notation.

### Data value constraints mapping

The goal of this step is to map the data types and associated constraints that may occur within archetypes to those supported by the EHR system model. The AM provides eight primitive types, which may be constrained by an archetype leaf node [26]. These primitive types have to be mapped to corresponding data types in the EHR system model.

### Terminology related mapping

The goal of this step is to map the terminology bindings within archetypes to the EHR system model. Archetypes may define locally defined terms and associated display strings as allowed value sets for their nodes. They may also define bindings for their nodes to terms in external terminologies within their *term_bindings* section.

### Generation of standardized and archetype-compliant EHR extracts from the collected documents

To prepare for the generation of standardized and archetype-compliant EHR extracts from the data collected via the generated forms, we record the complete path of the original comprehensive archetype node during the generation of each form component. This is necessary since, owing to the fact that the RM is typically more expressive than the EHR system data model [20], different RM classes will usually be mapped to the same EHR system data model class. Thus, the class of the original archetype node cannot be unambiguously recovered from the types of the generated form components.

Each time a generated form is populated with data, a document is created in the EHR system. For each document that needs to be exported as a standardized and archetype-compliant EHR extract, the underlying form components are retrieved. Based on the structure of the form and the paths of the comprehensive archetype nodes, which were recorded during the creation of the form components, the XML-based EHR extract is composed from the source document data. If the complete paths of the archetype nodes associated with each form component are stored instead of only the node identifiers, the structure of the EHR extract can be assembled without having to access the original archetype. In our prototype we stored the paths of the archetype nodes in XPath format.

To export data from a legacy EHR system as openEHR conformant EHR extracts, the *Generic_extract* package of the Extract Information Model specification [27] must be used. Existing XML schemas [28] of the openEHR RM and the Extract Information Model may be used to validate the EHR extract.

## Results

In the following, we present our prototype implemented within the EHR system ArchiMed. For ease of explanation, we refer to an example, which shows how an ArchiMed form (see Figure 3) is automatically generated from the archetype depicted in Figure 2.

### Automatic generation of ArchiMed forms from OpenEHR archetypes

As part of our prototype we used the open source Java-version of the archetype parser that was developed in the course of the openEHR Java Reference Implementation Project [29] and is available from [25].

### Structural constraints mapping

**Figure 6:**
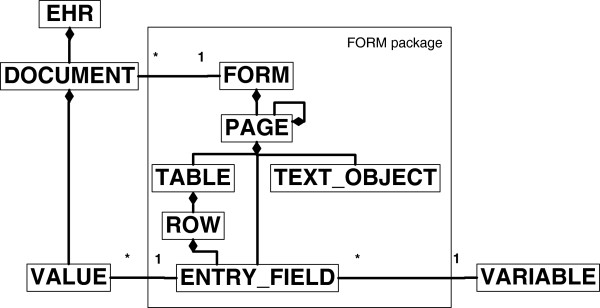
**Simplified example of the ArchiMed data model in UML format.** In the ArchiMed system an EHR consists of a set of DOCUMENTs, which themselves hold data VALUEs. The possible DOCUMENT structures are determined by the FORM package within the data model. One instance of the FORM class describes the structure of a class of DOCUMENTs. Each time a FORM is populated with data for a particular patient, the patient data are stored in a new DOCUMENT for this particular FORM. A FORM consists of one or more PAGEs. PAGEs may contain other PAGEs, TEXT_OBJECTs (e.g., fixed text, lines, boxes), or ENTRY_FIELDs. Each ENTRY_FIELD refers to a VARIABLE (e.g., systolic blood-pressure), and holds an actual data VALUE. VARIABLEs may be reused by different ENTRY_FIELDs. For VALUEs only primitive data types are supported. If multiple VALUEs need to be collectable for an ENTRY_FIELD, the latter may be embedded in a TABLE. A TABLE may also nest a group of logically related ENTRY_FIELDs.

Figure 6 shows the data model of the ArchiMed system. Data entry forms are represented by the class FORM in ArchiMed, which is thus the target of the root node in our mapping. In Figure 2 the root node *at0000* of the archetype is an OBSERVATION named “Body mass index”.

Archetype leaf nodes are mapped to ArchiMed ENTRY_FIELDs. If a leaf node prescribes a list of predefined terms, they are offered as choice lists within the ENTRY_FIELD.

The ArchiMed data model includes the “organisational” components PAGE and TEXT_OBJECT. We use the former to represent archetype SECTION nodes. Intermediate archetype nodes are mapped to TEXT_OBJECTs. This ensures that the context of the leaf nodes as defined in the archetype is depicted in the form.

Repeating archetype nodes (such as node *at0002* in Figure 2) are mapped to ROWs of a TABLE. The TABLE is named after the repeating node. The leaf nodes of the different branches “below” the repeating node represent the columns of the TABLE. The names of the nodes below the repeating node down to the leaf nodes are concatenated to form the labels of the TABLE columns. As an example, the leaf nodes of node *at0002* are given by attributes *property*, *units*, *magnitude* and *precision* of node C_DV_QUANTITY, which all receive the prefix “Single.BodyMassIndex.value” in their label according to the names of their superordinate nodes. Attribute *property* is no longer supported in the current version of class DV_QUANTITY [30] and is thus ignored. Attribute *units* is represented as a textual column, with the two alternatives for its value offered as a choice list.

Additional ArchiMed ENTRY_FIELDs are created for all mandatory attributes of the RM that were added in the creation of the comprehensive archetype (e.g., HISTORY’s attribute *origin* and EVENT’s attribute *time* as shown in Figure 3).

As ArchiMed forms may not contain subforms, archetypes included via slots are expanded in the including archetype. In the case of “wildcard” slots, the list of allowed archetypes has to be narrowed down to a single archetype. Similarly, internal archetype references are expanded at the referring locations.

### Data value constraints mapping

Table 1 depicts how the eight primitive types, which may be constrained by an archetype leaf node, are mapped to ArchiMed’s data types. Owing to ArchiMed’s limited set of data types, some of the primitive types are mapped to more generic types. As an example, Boolean values are stored as textual values “Y/N” in ArchiMed, as the underlying Oracle database does not support Boolean data types. Within ArchiMed forms, ENTRY_FIELDs of this kind are visualized as checkbox widgets to ensure two-valued inputs. During the generation of the EHR extract the internal values are then transformed to the Boolean values “true/false”.

**Table 1:** Mapping of archetype primitive types to ArchiMed data types

**Archetype primitive type**	**ArchiMed data type**
Boolean	Text
String	Text
Integer	Number
Real	Number
Date	Date
Time	Time
Date_Time	Timestamp
Duration	Text

ArchiMed only supports a subset of the archetype data type constraints. Some constraints can be partly represented, such as date and time pattern constraints through different formats for ENTRY_FIELDs. Others cannot be represented, including duration pattern, regular expression, and assertion constraints.

### Terminology related mapping

Analogous to the AM, ArchiMed allows ENTRY_FIELDs to be parameterized in a way that they only accept VALUEs from a predefined set of terms and display strings. The AM’s *term_bindings* section cannot be represented within ArchiMed, as the system currently does not support form components that are annotated with terms additional to their labels. The local terms of archetypes nodes are used to label the corresponding form components.

### Generation of standardized and archetype-compliant EHR extracts from the collected documents

**Figure 7:**
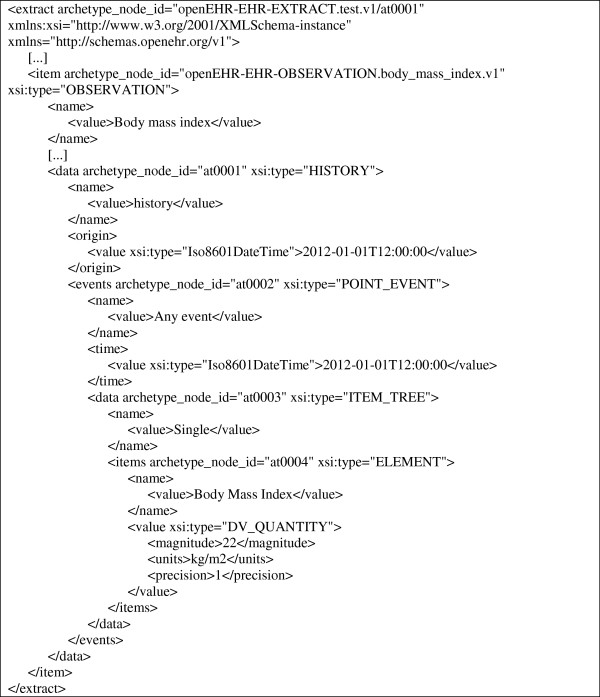
**Excerpt of an EHR extract created from data recorded via the form depicted in Figure**3**.**

For the generation of standardized and archetype-compliant EHR extracts we implemented a PL/SQL procedure in ArchiMed’s Oracle database. For each ArchiMed document that was collected via an archetype-derived form and needs to be exported as an EHR extract, it retrieves the underlying form components. Based on the structure of the form and the paths of the archetype nodes, which were stored in XPath format for the form components (cf. Table 2), the XML-based EHR extract is composed from the source document data. Figure 7 shows an example of an EHR extract that was generated from data recorded via the form depicted in Figure 3.

**Table 2:** **Sample metadata entries of selected form components depicted in Figure**3

**Type**	**Label**	**Path**
PAGE	Body mass index	[@archetype_node_id = ‘openEHR-EHR-OBSERVATION.body_mass_index.v1’ and @xsi:type = ‘OBSERVATION’]
TEXT_OBJECT	history	[@archetype_node_id = ‘openEHR-EHR-OBSERVATION.body_mass_index.v1’ and @xsi:type = ‘OBSERVATION’]/data[@archetype_node_id = ‘at0001’ and @xsi:type = ‘HISTORY’]
TEXT_OBJECT	origin	[@archetype_node_id = ‘openEHR-EHR-OBSERVATION.body_mass_index.v1’ and @xsi:type = ‘OBSERVATION’]/data[@archetype_node_id = ‘at0001’ and @xsi:type = ‘HISTORY’]/origin[@xsi:type = ‘DV_DATE_TIME’]
ENTRY_FIELD	value	[@archetype_node_id = ‘openEHR-EHR-OBSERVATION.body_mass_index.v1’ and @xsi:type = ‘OBSERVATION’]/data[@archetype_node_id = ‘at0001’ and @xsi:type = ‘HISTORY’]/origin[@xsi:type = ‘DV_DATE_TIME’]/value[@xsi:type = ‘DATE_TIME’]

Column *Type* depicts the ArchiMed data model class corresponding to the particular archetype node. Column *Label* holds the component’s label in the form. Column *Path* holds the path of the archetype node (in XPath format), from which the form component is derived. Components *origin* and *value* are not addressed in the archetype, but have to be considered in the form and the EHR extract as they are mandatory attributes of the RM.

### Archetypes integrated into ArchiMed

We used 27 existing archetypes in our study (cf. Table 3), which we drew from the openEHR online archetype library [23]. The archetypes were selected on the following basis: At the time of our study a total of 224 archetypes existed in the repository. After removing the archetypes with status “draft”, 16 archetypes remained with status either “published” or “team review”. These archetypes formed our basic test set. The test set had to be augmented with the 11 additional archetypes to which the original 16 archetypes referred within archetype slots.

**Table 3:** Archetypes selected for integration in the ArchiMed system in this study

**No.**	**Archetype ID**	**Integration successful**
1	openEHR‐EHR‐CLUSTER.macroscopy_colorectal_carcinoma.v1	Yes
2	openEHR‐EHR‐CLUSTER.microscopy_breast_carcinoma.v1	No
3	openEHR‐EHR‐CLUSTER.microscopy_lung_carcinoma.v1	No
4	openEHR‐EHR‐CLUSTER.microscopy_lymphoma.v1	Yes
5	openEHR‐EHR‐CLUSTER.microscopy_melanoma.v1	No
6	openEHR‐EHR‐CLUSTER.microscopy_prostate_carcinoma.v1	No
7	openEHR‐EHR‐CLUSTER.tnm_staging‐lung_cancer.v1	Yes
8	openEHR‐EHR‐EVALUATION.adverse.v1	Yes
9	openEHR‐EHR‐EVALUATION.clinical_synopsis.v1	Yes
10	openEHR‐EHR‐OBSERVATION.apgar.v1	Yes
11	openEHR‐EHR‐OBSERVATION.blood_pressure.v1	No
12	openEHR‐EHR‐OBSERVATION.body_mass_index.v1	Yes
13	openEHR‐EHR‐OBSERVATION.body_temperature.v1	No
14	openEHR‐EHR‐OBSERVATION.body_weight.v1	No
15	openEHR‐EHR‐OBSERVATION.height.v1	No
16	openEHR‐EHR‐OBSERVATION.respiration.v1	No
17	openEHR‐EHR‐CLUSTER.ambient_oxygen.v1.adl	Yes
18	openEHR‐EHR‐CLUSTER.anatomical_location‐precise.v1.adl	Yes
19	openEHR‐EHR‐CLUSTER.device.v1.adl	No
20	openEHR‐EHR‐CLUSTER.environmental_conditions.v1.adl	Yes
21	openEHR‐EHR‐CLUSTER.level_of_exertion.v1.adl	Yes
22	openEHR‐EHR‐CLUSTER.lymph_node_metastases.v1.adl	Yes
23	openEHR‐EHR‐CLUSTER.physical_properties.v1.adl	Yes
24	openEHR‐EHR‐CLUSTER.tumour_invasion.v1.adl	No
25	openEHR‐EHR‐CLUSTER.tumour_resection_margins.v1.adl	No
26	openEHR‐EHR‐ELEMENT.last_normal_menstrual_period.v1.adl	Yes
27	openEHR‐EHR‐ELEMENT.menstrual_cycle_day.v1.adl	Yes

Of this test set, 15 archetypes could be successfully integrated in the ArchiMed system. For 12 archetypes the form generation failed. In all cases the problem was caused by multiple repeating archetype nodes, i.e., a node with an upper occurrence limit greater than 1, which contains another repeating subnode. This problem is explained in more detail in the discussion section.

## Discussion

Although we have thus far only tested our approach with the openEHR architecture, the extension to the ISO/EN 13606 architecture seems to be rather straight-forward. Archetypes are modelled identically in both standards. Even though they use different RMs (which are actually similar), the archetypes’ properties that are relevant to our approach are the same:

● Analogously to openEHR, 13606 archetypes only include those constraints, which they tighten with respect to the RM. Thus, the creation of comprehensive archetypes is once again required.

● Repeating nodes also exist in 13606 archetypes, as the 13606 RM includes several *one too many* relations that may be left unconstrained by the archetype.

The architectures differ in the data types they use and in the way an EHR extract is modelled. Therefore, a specific mapping from the 13606 data types to the EHR system data types would have to be defined. Further, the EHR extract generator would have to be adapted to the 13606 model of EHR extracts.

Alternatively, existing approaches for EHR standards transformation could be used to apply our method in an ISO/EN 13606 environment. Here, the 13606 archetypes to be integrated in the EHR system would be transformed to openEHR archetypes [31]. The generated openEHR EHR extract could then be transformed back to the 13606 format [32].

A problem that we experienced was that the high level of optionality in archetypes complicates the direct derivation of EHR forms. This optionality is a consequence of the typical “maximal data set” design of archetypes, in which all components that may be relevant in any possible scenario, are included. This often leads to forms that are overburdened with entry fields that are not needed in a particular application context.

A high level of optionality may even preclude the generation of a corresponding form, if the EHR system’s data model does not support the complex structures prescribed by the archetype. In the ArchiMed system, this was the case when multiple levels of repeating nodes, i.e., repeating nodes containing other repeating subnodes (cf. Figure 4) were specified by the archetype. Such nodes would have to be represented by means of recursive TABLEs, which ArchiMed currently does not support.

To avoid this problem, archetypes should be customized for the current application context, before deriving forms from them. In these customized archetypes all irrelevant optional nodes could be removed, while optional complex structures, such as multiple levels of repeating nodes, could be dissolved.

For this purpose, the openEHR architecture envisages so-called “templates” as a second constraint layer on top of archetypes. Among other purposes, they are also intended to be used as a basis for form generation [33].

We decided to base our approach on archetypes instead of templates for the following reasons:

● Templates are currently only considered in openEHR, and even there the specification still has the status “Development”. ISO/EN 13606 does not support templates.

● OpenEHR templates are defined as specialized archetypes and formally expressed in terms of the AM [33]. All characteristics of an archetype, on which our approach is based, apply equally to an openEHR template. Thus, all insights gained through our approach are still valid and have to be considered when generating forms from openEHR templates.

Consequently, an archetype-based approach shares common ground with ISO/EN13606 and at the same time remains compatible with the upcoming openEHR templates specification.

Within the ISO/EN 13606 architecture, the cus-tomization of archetypes for the purpose of form generation could be achieved via specialized archetypes. Here, irrelevant optional nodes of the parent archetypes could be removed and multiple levels of repeating nodes could be dissolved.

As the COSMIC and ArchiMed studies show, the data types and associated constraints as well as the terminology bindings provided by the EHR system model are likely to be less extensive than those defined in the AM. Some archetype data types will then have to be mapped to more generic data types in the EHR system model. For several archetype data type constraints and terminology bindings, there may even be no corresponding functionality in the EHR system model.

Such gaps in the EHR system’s data model may result in a loss of data quality, if the EHR system data type cannot natively enforce a particular constraint defined in an archetype. Missing constraints may partly be enforced at the application level, such as our implementation of Boolean values in ArchiMed. In some cases, gaps may be acceptable. As an example, even though the archetype *term_bindings* section cannot be represented in ArchiMed, we considered the labelling of ArchiMed form components by means of the archetype local terms to be sufficient for our purposes. For certain types of gaps, however, the relevant archetypes may be considered outside of the EHR system’s “modelling safe zone” [20].

In the creation of comprehensive archetypes, we only considered unconstrained *mandatory* attributes of the RM for inclusion in the generated form. Optional attributes were assumed not to be needed for data collection as they were not set to mandatory in the archetype. This assumption allowed us to limit the number of optional archetype nodes in a rudimentary way and thus also the size of the generated forms. It can, however, be argued that unconstrained optional attributes may nonetheless need to appear in the form. In this case, the comprehensive archetype would simply have to be extended to include these attributes as well. Interestingly, the current openEHR template designer [34] offers neither optional nor mandatory RM attributes to the user if they are not constrained by the underlying archetype. Nor do they appear in the existing Operational Templates published in [23]. Even though they can be added during form generation, the user cannot specify in the template whether a particular attribute should be included in the form.

Our approach is limited insofar as immediate generation of EHR extracts is only possible for documents that were recorded via the archetype-derived forms. In this case, the mapping between the form contents and the EHR extract is automatically generated during creation of the form. Data recorded otherwise, which should be included in the EHR extracts, would either have to be loaded in the database tables storing data for the archetype-derived forms, or be manually mapped to the corresponding archetype nodes.

We examined in a lab environment to what extent archetype data structures could be represented within EHR system forms. To be applicable in clinical practice, the usability of the generated forms has to be improved. To achieve a high level of usability, a separate visualization knowledge layer could be added that specifies a suitable way of presenting each archetype node in a form. Van der Linden et al. presented a corresponding approach that distinguishes content-related, localized, and device-related presentation knowledge [35]. Kopanitsa proposed a presentation layer that considers different views for different users and devices [36], while Atalag et al. relied on a set of graphical user interface directives to adjust the aesthetics and visual behaviour of generated forms [37].

## Conclusions

In this paper we presented an approach for plug-and-play integration of archetypes in legacy EHR systems, which (a) automatically generates entry forms in the EHR system from archetypes, and (b) supports immediate export of EHR documents that are recorded via the generated forms and stored in the EHR system’s internal proprietary format as standardized and archetype-compliant EHR extracts. We specified a set of basic requirements, which an EHR system’s data model must satisfy, as a prerequisite for applying our approach.

We tested our approach with the ArchiMed system and were able to successfully integrate 15 archetypes from a test set of 27. For 12 archetypes, the form generation failed owing to multiple levels of repeating nodes resulting from the typical “maximal data set” design of archetypes.

To avoid this problem and further reduce the derived form to those entry fields that are relevant in a particular application context, archetypes should be customized before generating the form. This may be done by means of openEHR templates or by specializing archetypes. In both cases, complex structures such as multiple levels of repeating nodes could be dissolved and irrelevant archetype nodes could be removed. We intend on employing these variants of archetype customization in two upcoming projects, where we aim to integrate openEHR respective ISO/EN 13606 archetypes in legacy EHR systems.

Compared with archetypes, an EHR system data model is often less expressive, in particular with regard to supported data types and terminological features. Some of the resulting gaps may be closed by workarounds at the application level and some may be acceptable in a given application scenario. For the remaining gaps, the relevant archetypes will have to be considered outside of the particular EHR system’s “modelling safe zone” [20], and thus cannot be integrated in the system.

Our results further show that the usability of the generated forms must be improved before they can be applied in clinical practice. To achieve this, approaches based on a separate visualization knowledge layer for archetypes may be employed [35-37].

## Competing interests

The authors declare that they have no competing interests.

## Authors’ contributions

GD conceptualized the proposed approach, and drafted as well as revised the manuscript. JC implemented the form generation approach for the ArchiMed system and assisted in revising the manuscript. CR implemented the transformation of EHR system documents to EHR extracts, contributed to the implementation of the form generation approach, and assisted in revising the manuscript. All authors read and approved the final manuscript.

## Pre-publication history

The pre-publication history for this paper can be accessed here:

http://www.biomedcentral.com/1472-6947/13/11/prepub
